# Blocking facial mimicry during binocular rivalry modulates visual awareness of faces with a neutral expression

**DOI:** 10.1038/s41598-021-89355-5

**Published:** 2021-05-11

**Authors:** Thomas Quettier, Filippo Gambarota, Naotsugu Tsuchiya, Paola Sessa

**Affiliations:** 1 Department of Developmental and Social Psychology, University of Padua, Via Venezia 8, 35121 Padua, Italy; 2 School of Psychological Sciences, Monash University, Clayton, Australia; 3 Padova Neuroscience Center (PNC), University of Padua, Padua, Italy

**Keywords:** Psychology, Human behaviour

## Abstract

Several previous studies have interfered with the observer’s facial mimicry during a variety of facial expression recognition tasks providing evidence in favor of the role of facial mimicry and sensorimotor activity in emotion processing. In this theoretical context, a particularly intriguing facet has been neglected, namely whether blocking facial mimicry modulates conscious perception of facial expressions of emotions. To address this issue, we used a binocular rivalry paradigm, in which two dissimilar stimuli presented to the two eyes alternatingly dominate conscious perception. On each trial, female participants (N = 32) were exposed to a rivalrous pair of a neutral and a happy expression of the same individual through anaglyph glasses in two conditions: in one, they could freely use their facial mimicry, in the other they had to keep a chopstick between their lips, constraining the mobility of the zygomatic muscle and producing ‘noise’ for sensorimotor simulation. We found that blocking facial mimicry affected the perceptual dominance in terms of cumulative time favoring neutral faces, but it did not change the time before the first dominance was established. Taken together, our results open a door to future investigation of the intersection between sensorimotor simulation models and conscious perception of emotional facial expressions.

## Introduction

Recognizing others’ emotions and affective states is one of the most extraordinary human abilities. In a purely evolutionary perspective, detecting and understanding—quickly and accurately—others’ emotions, desires and intentions clearly offers adaptive advantages and promotes affiliation, mating and parenting. For a long time, psychological and neuroscientific research has explored the cognitive and neural bases of this ability^1–7^. Recent neuroimaging research has provided converging evidence that the brain networks for facial expressions processing comprise several cortical and subcortical regions. These include the fusiform face area, the occipital face area, the superior temporal sulcus (regions of the core system of Haxby’s distributed model of face processing^8–15^), the insula, the amygdala, the inferior frontal gyrus^16–21^, and several other not strictly face-sensitive regions^22^.

In this context, motor (or sensorimotor) simulation models propose that the observer’s subthreshold motor simulation of the observed facial expression facilitates recognition and understanding of others’ congruent facial expression^23–25^. This theoretical view is supported by a substantial body of evidence. On one side it has been highlighted how brain regions supporting motor and somatosensory representations of facial expressions are involved in recognition of emotions in others^26^, such that, for instance, lesions of these regions are associated with emotion recognition deficits^7,27^ and, similarly, repetitive transcranial magnetic stimulation disrupting the right somatosensory^6, 28^ and the right primary motor^29^ cortices impairs some aspect of emotional face processing; on the other side there is evidence that muscular facial feedback incongruent with the observed expression causes a decrease in emotion recognition accuracy^26,30–36^, (but also see^37–40^). The rationale behind this last series of studies is that, if facial mimicry—which can be measured by electromyography^41,42^—is a manifestation of sensorimotor simulation triggered by the observation of others’ facial expressions, then an experimental manipulation aimed at interfering with it should consequently interfere with the simulation process itself and thus affecting the processing of facial expressions. For example, Ponari et al. (Experiment 1^35^) required participants to identify the emotion (among the basic six categories) expressed by the faces presented one at a time while the production of their facial mimic patterns was manipulated through a Chinese chopstick (which participants had to keep horizontally between the teeth to prevent the movements of the lower portion of the face) or through two stickers near the inner portion of the eyebrows (which participants had to actively try to bring near through an active contraction of the frontal muscles). The results highlighted an impairment of the accuracy in the identification task as a function of the type of facial mimicry manipulation, i.e., that interfering with the muscles of the lower portion of the participants’ faces was associated with an impairment in the processing of happiness, disgust and fear, while that interfering with the muscles of the upper portion of the face was associated with an impairment of the processing of anger and fear.

While the behavioral evidence is accumulating to support the role of sensorimotor activity and facial mimicry in facilitating the recognition of others’ congruent emotional expressions, it remains unclear at what level of the visual processing of emotional faces the sensorimotor activity might exert its influence^43,44^. In this regard, a recent sensorimotor simulation model has proposed that sensorimotor signals may feedback to visual areas modulating the visual processing of emotional faces from early stages^26,45^.

Notably, in these theoretical and empirical frameworks an aspect that has been almost totally neglected is that concerning the possible role of facial mimicry in conscious perception of emotional facial expressions. The present study aimed at investigating precisely this aspect of facial mimicry/sensorimotor simulation using binocular rivalry, a paradigm widely used in the studies of visual conscious perception and its neural correlates^46–49^.

Under the ecological circumstances, slightly discordant visual inputs to the two eyes result in stable stereo experience (but also see^50^). When the dissimilarity exceeds a certain threshold, periods of perceptual dominance of one stimulus over the other stochastically alternate, such that one monocular image is dominantly consciously experienced while the other is suppressed and invisible^47,51^. This condition is called binocular rivalry (BR)^52^.

**Figure 1:**
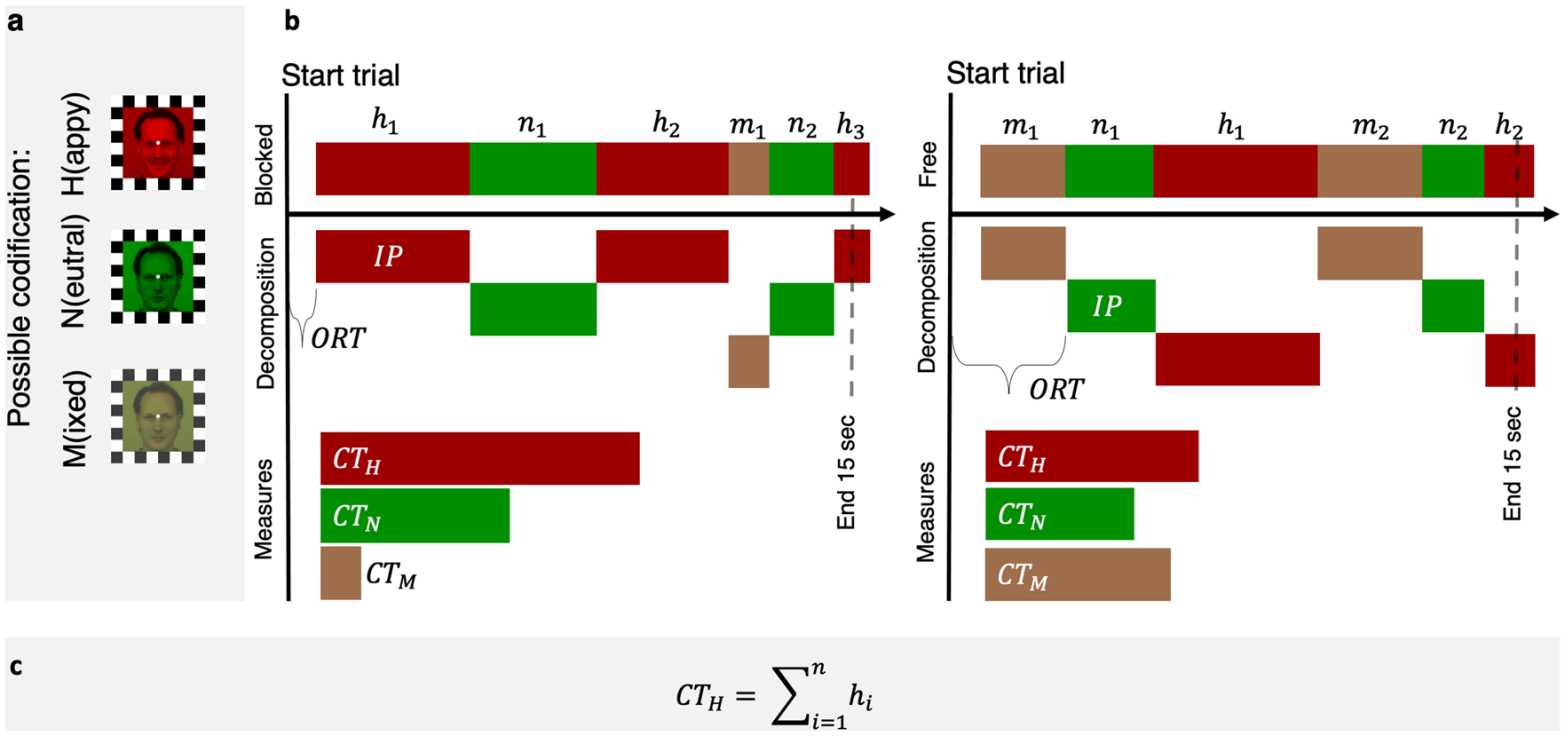
Rivalry during trials and BR measures. (**a**) Possible coding of the rival stimuli. Original pictures (AM10NES AM10HAS) of facial expressions have been selected from the Karolinska Directed Emotional Faces set (KDEF; https://www.kdef.se/) and modified using GIMP (version 2.8.10, https://www.gimp.org). (**b**) An example of a subject rivalry trials and coded percepts (e.g., h1: first happy face coded in a trial, n1: first neutral face coded in a trial, m1: first mixed percept coded in a trial…). The three behavioral measures that we extract from this time course are (1) initial percept (IP) (either Happy, Neutral), (2) onset resolution time (ORT in [s]), and (3) cumulative time (CT for percept in [s]), formula in (**c**).

In the BR paradigm, participants are often required to report their content of consciousness in a continuous manner among several alternatives (see Fig. 1). The time series data of BR report is quite rich and has been dissected into at least three components, each of which can characterize the underlying neural and psychological processes from different perspectives. First, *initial percept* measures which of the rivaling stimuli dominates first in conscious percept, potentially characterizing any bias or advantage of one stimulus over the other. Second, *onset resolution time* measures the time of first *initial percept* button press, characterizing how long it takes the visual system to resolve perceptual ambiguity. In other words, it is an estimate of the time necessary for the brain to select (or inhibit) one percept over the other. These two BR components indicate the “winner” percept (i.e., initial percept) in the initial phase of unresolved competition (i.e., onset resolution time). A third component, which we call *cumulative time (CT)*, measures instead the total time of dominance in awareness of one percept over the other, i.e. the periods of relatively stable resolution following the initial competition.

Previous research has used the BR paradigm to study conscious perception of emotional stimuli, but rarely distinguished the three components of the BR described above. For example, some focused on how emotional faces predominate over neutral faces in BR^53–56^. Yoon et al.^56^, expanding on the research by Alpers and Gerdes^53^, demonstrated that emotional faces, regardless of their valence, are perceived for longer than neutral faces, although the effect was particularly strong (89% increase in dominance) for positive facial expressions (i.e., happy) which predominated also over negative facial expressions (i.e., disgusted). Unfortunately, the authors did not report the other two measures and the data are not openly available for further research.

Crucially, it is unknown if observer’s facial mimicry can influence conscious perception of facial expressions in the BR paradigm. We propose that there can be at least two possible ways, which are not mutually exclusive, to affect the BR. First, facial mimicry can influence the initial ambiguity resolution. Second, facial mimicry may stabilize such representations once they are the current content of consciousness. The present experiment aimed at investigating precisely this aspect of facial mimicry using BR under facial mimicry manipulation with a neutral and an emotional (i.e., happy) expression of the same identity in rivalry. We tested the impact of facial mimicry manipulation on both ambiguity resolution (i.e., initial percept and onset resolution time) and stabilization of conscious contents (i.e., cumulative time). We manipulated participants’ facial mimicry by means of a chopstick to be held firmly between the lips for half the experiment, while in the other half the facial mimicry was free (the order of the two mimicry conditions was counterbalanced between participants; for similar manipulations see^34,36,44,45,57,58^). This use of the chopstick inhibits the activation of the zygomatic major, associated with mimicry of facial expression of happiness^34,59^.

With regard to mimicry manipulation, only one previous study^60^ has investigated the impact of the integration of proprioceptive information from the face on visual awareness of facial expression of emotions using a variant of BR, called breaking continuous flash suppression (b-CFS^61–63^). In the b-CFS paradigm, a mask with a high contrast dynamic pattern is presented to one eye, thereby effectively suppressing a stimulus of increasing intensity presented to the other eye. After a certain interval of time, the ocular dominance is reversed, and the previous suppressed stimulus becomes visible. Thus, this paradigm measures the time for a stimulus to access consciousness. In a series of three experiments, Korb and colleagues manipulated participants’ facial mimicry using a b-CFS paradigm by requiring them to voluntarily take facial expressions of frown vs. smile vs. relaxed (Experiments 1 and 2) or by measuring spontaneous electromyographic activity (Experiment 3). Overall, the results did not support the hypothesis of a modulation of visual consciousness on the basis of the integration of the proprioceptive activity of the face in conditions of congruence and incongruence with the observed emotion. That is, the time to break suppression did not differ between conditions. This suggests that facial mimicry does not influence the access to consciousness of emotional facial expressions. To note, b-CFS cannot inform on the potential role of facial mimicry in stabilizing conscious representations of facial expressions.

We expected that blocking/altering facial mimicry could interfere with the alternation in BR. In particular, we expected that interfering with the sensorimotor signal (i.e., by means of the active inhibition of facial mimicry) would (1) favor the neutral facial expressions and/or (2) interfere with happy expressions with respect to the condition of free mimicry by either biasing competition and ambiguity resolution at the early stage and/or stabilizing representations following initial resolution at the late stage. This last scenario seems more plausible in the light of the results reported by Korb and colleagues. To test the early account (i.e., ambiguity resolution), we analyzed the frequency and time of the first rivalry report, i.e. initial percept and onset resolution time, respectively^64^. To test the late account (i.e., stabilization), we analyzed the cumulative time. As an auxiliary although interesting aim, we wanted to replicate the few interesting results previously reported in the literature about an advantage in terms of predominance of emotional stimuli, and in particular of facial expressions of happiness when in rivalry with neutral facial expressions^53,55,56^.

Since previous studies have suggested that facial mimicry manipulations have a greater impact on female than male participants^36,45^ (for compatible results see also^29^), we decided to recruit exclusively female participants in order to maximize power. Therefore, on the basis of the hypotheses introduced in the previous paragraph, from a statistical point of view, the effect that we expected, both for the tests of the early and the late accounts, was an interaction between facial expression and mimicry manipulation, with (1) an advantage for neutral expressions under the condition of blocked mimicry when compared to free mimicry, and/or (2) a disadvantage for happy expressions under the condition of blocked mimicry when compared to free mimicry.

Finally, on an exploratory basis, in the present investigation we also tested the hypothesis of a relationship between alexithymic traits and awareness of emotional expressions. Alexithymia is defined as a difficulty in recognizing one’s own and others’ emotions^65^. We hypothesized that the different binocular rivalry metrics we analyzed could show a relationship with the levels of alexithymic traits, in the direction of the decreasing in conscious perception for emotional expressions with increasing levels of alexithymia.

## Results

### Ratings

Evaluation of valence and arousal were performed on individual stimuli at the end of each session. No effect of the session in terms of mimicry manipulation was observed (valence *F*(1, 27) = 2.28, *p* = 0.143; arousal *F*(1, 27) = 0.08, *p* = 0.775). Valence ratings differed according to a priori expectations, *F*(1, 27) = 330.02, *p* < 0.001. Neutral facial expressions were rated close to zero (*M* = − 0.63; *SD* = 0.98; range = − 3 to 3), which was more negative than happy (*M* = 2.15; *SD* = 1.12; range = − 3 to 3). Happy expressions were rated more positively than neutral expressions, *t*(27) = 18.16 *p* < 0.0001. Arousal ratings also differed according to a priori expectations, *F*(1, 27) = 56.54, p < 0.001. They were lower for neutral expressions (*M* = 2.74; *SD* = 1.54; range = 1–7) than for happy expressions (*M* = 4.97, *SD* = 1.63; range = 1–7). Neutral facial expressions were rated as significantly less arousing than happy facial expressions, *t*(27) = 7.52, *p* < 0.0001.

### Testing the early effects of mimicry blocking (i.e., resolution of ambiguity)

For both initial percept (IP) and onset resolution time (ORT), the statistical models included the factors mimicry (free vs. blocked) and the first clear facial expression reported (i.e., IPs; happy vs. neutral), in line with the very definition of resolution of ambiguity between rivalrous percepts.

### Initial percept (IP)

In terms of IPs, happy expressions were reported more frequently in both mimicry conditions (blocked: 304 trials; free: 317 trials) than neutral expressions (blocked: 128; free: 113). The odds ratio is not statistically significant, and the estimated value is 0.83 (*β* = − 0.184, *SE* = 0.159, 95% CI [− 0.50, 0.13], *t* − 1.15, *p* = 0.248).

### Onset resolution time (ORT)

**Figure 2:**
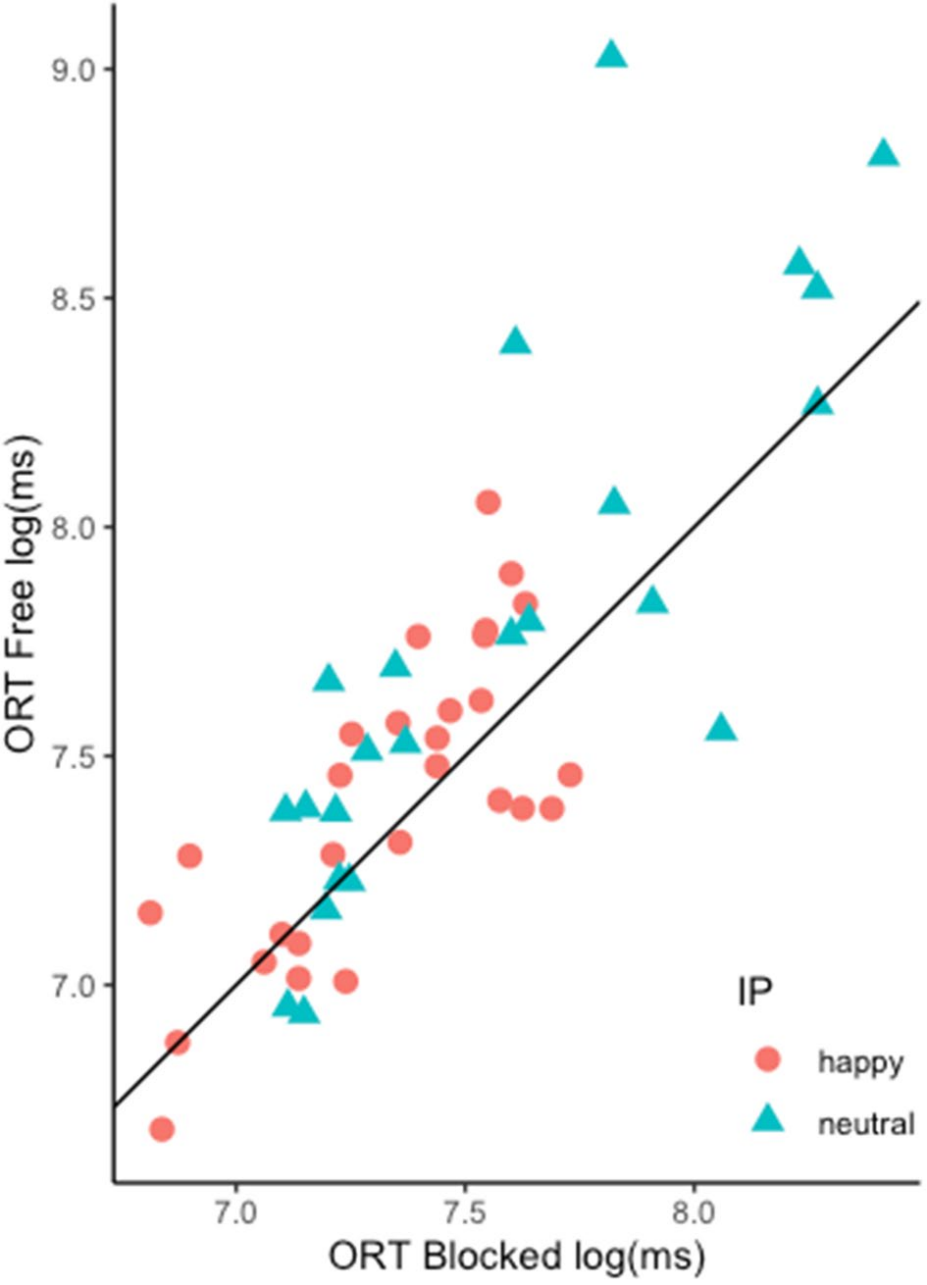
Each point represents a participant’s onset resolution time (ORT) for a specific IP. Mimicry condition is projected on the x and y axes, the black line represents the axes equidistance. The ORT is expressed in milliseconds.

Due to the IP distribution, five participants were removed from analysis (i.e., they did not have all possible IPs). Significant differences were found for the mimicry condition independently of the first clear facial expression reported (i.e., IPs), *F*(1,22) = 6.38, *p* = 0.019 and for the IPs independently of mimicry condition, *F*(1,22) = 13.90, *p* < 0.001. See Fig. 2.

In general, the findings related to the IP and ORT metrics seem not to support the early account, that is, the inhibition of the zygomatic muscle did not influence the resolution of ambiguity in favor of neutral faces and/or at the expense of happy facial expressions. These results seem to be in line with the previous evidence provided by Korb et al.^60^.

To note, in terms of IP frequency and ORT, emotional faces showed an advantage when compared to neutral faces irrespective of the mimicry conditions.

### Testing the late effects of mimicry blocking (i.e., stabilization of conscious contents)

For the cumulative time (CT) metric, the statistical model included the factors mimicry (free vs. blocked) and the reported content (happy vs. neutral vs. mixed).

**Figure 3:**
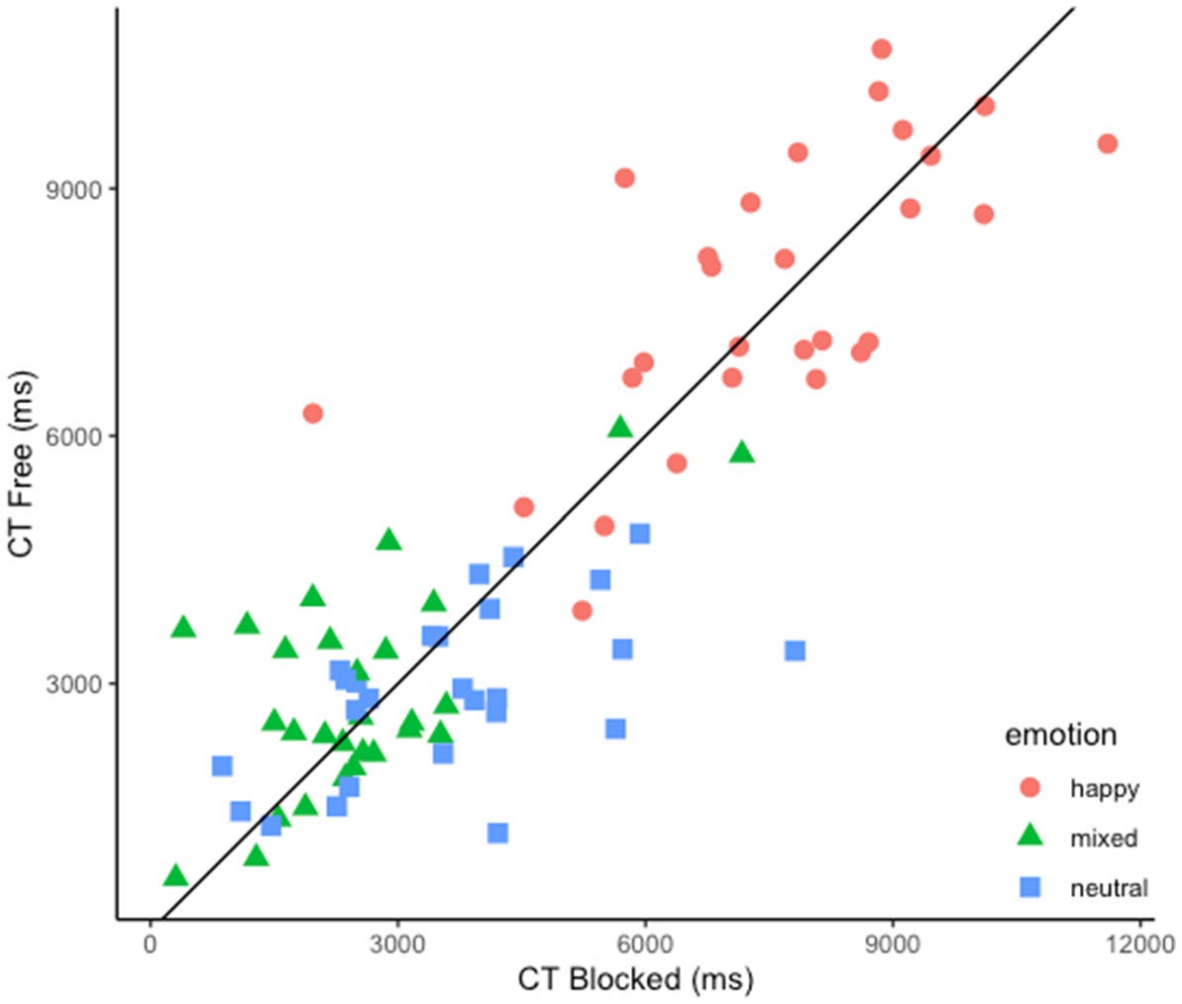
Each point represents a participant’s cumulative time (CT) for a specific emotion. Mimicry condition is projected on the x and y axes, the black line represents the axes equidistance. The CT is expressed in milliseconds.

A significant difference was observed for reported content independently of the mimicry condition, *F*(1.79, 48.26) = 69.18, *p* < 0.001. CT for happy facial expressions was longer than CT for neutral faces, *t*(54) = 9.78, *p* < 0.0001, and than CT for mixed percepts, *t*(54) = 10.54, *p* < 0.0001. This result is in line with previous findings^53, 56^. The main effect of mimicry did not yield to significant differences, *F*(1, 27) = 1.2, *p* = 0.283. In accordance with our hypothesis, CT for reported content showed an opposite trend as a function of the mimicry conditions, that is CT for neutral faces was expected to increase when mimicry was blocked (blocked mimicry condition: *M* = 3.39 s; *SD* = 4 s; free mimicry condition: *M* = 2.69 s; *SD* = 3.28 s), conversely CT for happy facial expressions was expected to decrease when mimicry was blocked (blocked mimicry condition: *M* = 7.51 s; *SD* = 4.96 s; free mimicry condition: *M* = 7.75 s; *SD* = 4.87 s). This observation was substantiated by the significant interaction between mimicry manipulation and the reported content, *F*(1.85,49.86) = 3.53, *p* = 0.04. However, our hypothesis was only partially supported by the post-hoc comparisons. In particular, the observed interaction was statistically conveyed entirely by a modulation of CTs for neutral expressions as a function of the mimicry manipulation, such that for neutral faces CT in the blocked mimicry condition was longer than CT in free mimicry condition, *t*(56.6) = 2.78, *p* = 0.007. The evidence in favor of the alternative hypothesis H1 may be classified, in terms of Bayes factor, as moderate, BF_10_ = 5.84. In order to substantiate our conclusions we further report the post-hoc comparisons for happy and mixed percepts (*t*(56.6) = − 0.928, *p* = 0.357, BF_10_ = 0.28; *t*(56.6) = − 1.348, *p* = 0.18, BF_10_ = 0.60, respectively). To note, the evidence in favor of the null hypothesis H0 for happy faces, can be classified as moderate, overall suggesting that the pattern of the observed findings is robust. See Fig. 3.

### Questionnaires

In exploratory analyses, we tested if ORT and CT were correlated (Pearson, two-sided correlations) with the Toronto Alexithymia Scale (TAS-20) and the Interpersonal Reactivity Index (IRI). No correlations were significant with the IRI scores. TAS-20 is one of the most commonly used measures of alexithymia traits, with a higher TAS-20 score indicating a higher inability to experience their own bodily emotions. Given that these tests were exploratory and since the study was not designed in terms of statistical power to directly answer this question on the relationship between alexithymic traits and BR metrics for facial expressions, we report the results without strict corrections for multiple comparisons, so that future studies can look into the promising correlations in a planned confirmatory testing.

TAS-20 score was negatively correlated with the cumulative time for the happy expressions in the free mimicry condition (i.e., CT_free_happy_; *r*(26) = − 0.42, *p* = 0.02), and positively correlated with onset resolution times for the happy expressions in the free mimicry condition (i.e., ORT_free_happy_; *r*(26) = 0.49, *p* = 0.007). The correlation with the cumulative time (i.e., CT_free_happy_) indicates that individuals with difficulty in experiencing their own emotion (as indicated by the high traits of alexithymia), tend to maintain emotional (here happy) faces as the content of their consciousness for a shorter time than individuals with lower levels of alexithymia. Furthermore, as indicated by the correlation with ORT (i.e., ORT_free_happy_), the initial time for perceptual disambiguation and conscious selection of an emotional face is greater in individuals with higher levels of alexithymia than in individuals with lower levels of alexithymia. This can be interpreted that alexithymic individuals tend to have difficulty and take longer time in resolving ambiguity in the direction of a more emotional face. It is interesting to note that these effects emerged in the condition in which participants could freely use their facial mimicry and these correlations are reduced in the blocked condition (*CT*_*blocked_happy*_: *r*(26) = − 0.23, *p* = 0.24 *ORT*_*blocked_happy*_: *r*(26) = 0.35, *p* = 0.06), which is along the direction that sensorimotor simulation theory would predict (see “Discussion”).

## Discussion

In the present investigation, we wanted to test the hypothesis of a functional role of the observer’s facial mimicry in ambiguity resolution and/or stabilization within awareness of faces with happy and neutral facial expressions during a binocular rivalry task. In particular, in the light of the sensorimotor simulation model by Wood et al.^45^, we hypothesized that the communication between sensorimotor and visual systems could either modulate the (initial) resolution of ambiguity under conditions of binocular rivalry or that signals from sensorimotor system could stabilize conscious representations of face stimuli in a later stage once the ambiguity is resolved. In order to test our hypothesis, we asked participants to perform a standard binocular rivalry task by presenting a happy and a neutral face (from the same identity) in rivalry. Crucially, we manipulated participants’ facial mimicry such that in one condition of the experiment they performed the task with their facial mimicry blocked by a chopstick to be held between the lips in order to inhibit the contraction of the zygomatic major, that is the muscle mainly involved when smiling; in the other half of the experiment participants could freely use their facial mimicry.

We reasoned that if the signal from the sensorimotor cortices is involved in the early conscious processing of facial expressions, inhibiting the zygomaticus major should have biased the initial competition between the two rivalry stimuli, such that initial resolution of the ambiguity would have been in favor of neutral faces in the blocked mimicry condition when compared to the free mimicry condition. Our primary outcome measures were the frequency of the initial percept (*IP*) and the onset resolution time (*ORT*) until the first rivalry as a function of the facial mimicry manipulation. A different but equally interesting scenario foresees that if the sensorimotor signal is integrated with the visual percept only at later stages of processing when the ‘winning’ stimulus is the content of consciousness, then blocking of facial mimicry would have modulated the stabilization of conscious perception of facial expressions. We tested this scenario through the analysis of the cumulative time (*CT*) as a function of the mimicry manipulation. We reasoned that, under an iterative model scenario (i.e., sensorimotor and visual systems that iteratively share information from very early stages of processing), an impact of mimicry manipulation could have been expected either on the initial percept and/or on the cumulative time.

First, and importantly, we replicated previous findings^53,56^ (see also^55^ for similar findings) such that cumulative time for happy facial expressions was longer than cumulative time for neutral faces, regardless of the mimicry manipulation condition. Furthermore, in our initial percept analysis, we found that happy expressions were more frequently perceived as first stimuli compared to neutral faces. These results corroborate the adequacy of the present paradigm and the quality of the present data.

Notably, the interaction between reported content (i.e., happy, neutral and mixed percepts) and mimicry manipulation did yield significant effects for the cumulative time. Further analyses provided evidence that cumulative time for neutral faces increased when mimicry was blocked compared to when participants could freely use their facial mimicry, thus providing only partial support to our hypothesis. In fact, although we observed a modulation of the cumulative time for neutral faces as a function of the mimicry manipulation, we did not observe an effect of the mimicry manipulation on the cumulative time for happy faces. Both of these results are corroborated by the Bayes factor.

How to reconcile these two results in the light of our initial hypothesis and the (sensorimotor) simulation models? A possible explanation could lie in the type of experimental manipulation of facial mimicry we have adopted here. In the condition of active alteration of mimicry (through the chopstick) it is possible that it was the *congruent cross-modal match* between the sensorimotor feedback and the visual representation of faces with neutral expression that has facilitated their stabilization in awareness. On the other hand, in our experimental design we did not implement a similar condition of facial mimicry congruent with the representation of happy expressions (e.g., by asking participants to actively keep the chopstick between their teeth, activating the zygomatic muscle). Future studies should clarify—possibly also including an electromyographic monitoring—whether it is this cross-modal congruence (sensorimotor-visual) that may facilitate stabilization in awareness for faces with different facial expressions, both neutral and emotional. An alternative explanation could rely on the notion that the simulation mechanism is especially triggered in those conditions in which facial expressions are particularly subtle or ambiguous (see, e.g.,^26^). Along this line of reasoning, neutral expressions may be conceived as more ambiguous than full expressions of happiness. Indeed, it is known that neutral expressions can be more easily misinterpreted than full emotional expressions, in both healthy^66^ and clinical populations^67–70^. In order to test this hypothesis, future studies could implement similar paradigms to that implemented here by varying the intensity of the rivalrous facial expression (for example through the morphing procedure). In light of these considerations, we admit that the present results are only generalizable to neutral facial expressions and to the role that facial mimicry can play in their stabilization in awareness. Nonetheless, we suggest useful future directions in order to investigate the phenomenon of the interaction between sensorimotor simulation and visual awareness that is currently almost totally neglected.

Another aspect that will require future work to provide a complete picture of the generalizability of these results concerns the female sample of the present study. In the light of the novelty of this field of investigation, we deemed appropriate including a sample of only women in order to maximize the probability of observing an effect because of the large body of previous evidence showing that women are more expressive than men^71^, more accurate in processing emotional expressions^72^, and selectively impaired in their ability to process expressions of happiness following the disruption of the primary motor cortex by repetitive transcranial magnetic stimulation^29^. Our results are currently generalizable to women and future experimental designs should include the participants’ gender as a factor.

Although a similar trend to that observed for the cumulative time was observed for the initial percept metric (i.e., a higher frequency for faces with neutral expression in the condition of blocked mimicry compared to the condition of free mimicry and an opposite trend for faces with expression of happiness), the effect was not statistically significant (when corrected for multiple comparisons). These findings indicate that the signal of the sensorimotor system tends not to play a role before the resolution of the ambiguity in favor of one of the two stimuli in rivalry, but rather that the signal from the sensorimotor system mainly acts as a ‘stabilizer’ of the conscious representation of the neutral face once the stimulus is the current content of consciousness (to note, the concept of ‘stabilization’ used here is not to be intended as for Leopold^73^ and for Pearson and Brascamp^74^).

At the present, this whole pattern of findings is almost entirely in line with the results previously reported by Korb et al.^60^ using a breaking continuous flash suppression (b-CFS) task, especially with regard to the early account we tested. However, and importantly, we observed an effect of the mimicry manipulation on the conscious stabilization of the neutral expressions, suggesting indeed a role of the sensorimotor system during a later stage of conscious processing which deserves to be investigated further, exploring the possibility that it can be generalized to emotional expressions.

Taking into account the line of studies on the neural correlates of consciousness and the debate on the temporal onset of consciousness (see, e.g.,^75^), it is interesting to note that two different temporal *loci* have been proposed, one “early” (associated in terms of event-related potentials, ERPs, with the visual awareness negativity in the range of the N2 ERP component^76–78^), and one “late” (associated with P3b/LP ERP component; e.g.^79^). Instead, as regards the effects of the blocking of facial mimicry on the construction of a visual percept, a previous study by our research group^44^ has shown an impact of the facial mimicry manipulation on visual working memory representations of neutral and emotional faces in terms of a modulation of the sustained posterior contralateral negativity ERP component detected at occipito-temporo-parietal sites (SPCN; e.g.^80–82^). To note (a) this ERP component has an onset of about 300 ms post-stimulus, thus supporting the view that visual and sensorimotor information may interact/combine within 300–400 ms following the exposure to a facial expression, and (b) the onset of this ERP component is later than visual awareness negativity (i.e., early temporal locus of the onset of consciousness) and earlier than the P3b/LP (i.e., late temporal locus of the onset of consciousness). Intriguingly, in another study designed to investigate the lower edge of this interaction between sensorimotor and visual systems, it was found that the ERP components P1 and N170 are not modulated as a function of facial mimicry manipulation, except in relation to alexithymic traits, in the direction of a modulation which tends to manifest itself only for individuals with low alexithymic traits^43^. An attempt to interpret this whole pattern of results could indicate that facial mimicry manipulations may affect high-level visual processing of facial expressions (approximately) after 170 ms and before 300–400 ms. The results of the present study, on the other hand, indicate that blocking of facial mimicry does not have an effect on the early resolution of perceptual ambiguity but rather on later stabilization of representations within awareness, at least for neutral facial expressions. Taken together, these results may suggest that the integration of somatosensory information with visual information occurs after the onset of awareness for facial expressions, thus suggesting an early onset of visual awareness. Considering the different time scales of the investigative phenomena (i.e., binocular rivalry and ERP findings), we propose this interpretation with great caution as a possible starting point for future studies.

Finally, although exploratory, we observed noteworthy correlations between the TAS-20 scores with metrics of binocular rivalry as a function of the mimicry manipulation. Correlation between TAS-20 scores and onset resolution time suggests that highly alexithymic participants tend to be slower in consciously accessing happy facial expressions when their facial mimicry is free and furthermore correlation with cumulative times indicated, instead, that individuals with higher traits of alexithymia under conditions of free mimicry tend to consciously perceive happy facial expressions for a shorter time compared to individuals with lower traits of alexithymia. These findings are perfectly in line with the very definition of alexithymia which is a subclinical condition involving a lack of emotional awareness and a difficulty in identifying (and describing) feelings and facial expressions of emotions^65^. We do not expand on the subject as the correlations we have investigated are currently exploratory, however we believe that this relationship deserves future investigation.

To conclude, the present results support an effect of facial mimicry on the awareness of faces with a neutral expression, in particular with regard to the stabilization of their conscious representations. However, we also acknowledge the need for future studies to investigate in greater depth the role of sensorimotor simulation on the conscious processing of emotional expressions and to consider both detecting facial electromyographic responses during a binocular rivalry paradigm similar to that employed in the present investigation, and, by means of time-resolved techniques such as electroencephalography, to track the time-course of the interaction between sensorimotor and visual cortices. In particular the latter would provide a more direct test of the early vs. late interaction hypotheses.

## Methods

### Participants

Thirty-two female healthy participants were recruited among students at the University of Padova (average age in years = 24.7, *SD* = 4.8, 2 left-handed). The sample size is considered appropriate on the basis of a meta-analysis on facial feedback and emotional experience^83^. Given the methodological heterogeneity, we selected a subsample of the originally included studies with these characteristics (a) facial mimicry manipulated as a within-subjects factor and (b) happiness as the main emotion. Using Coles et al.^83^ approach, the estimated effect in terms of Cohen’s *d*^84^ is 0.478 (*SE* = 0.162, 95% CI [0.117 0.839]). Power was estimated using the *pwr* package^85^ considering a paired t-test between blocked and free mimicry conditions. For the estimated effect size, a sample size of 30 participants is required to reach an 80% power level (see the online repository for additional details). Since we have hypothesized a specific direction of the effect due to mimicry, in this case we could consider a one-sided paired t-test, which would need a sample size of 28 participants to reach an 80% power level.

All volunteers gave written informed consent in accordance with the Declaration of Helsinki, and all experimental procedures were approved by the local research ethics committee (Comitato Etico della Ricerca Psicologica Area 17, University of Padua) and performed in accordance with its guidelines. Participants had normal or corrected-to-normal vision. Color blindness was assessed using the Ishihara color blindness test^86^. A total of four participants were excluded from analysis (one did not follow the coding instruction, two failed in holding the chopstick without the use of teeth and one did not complete the experiment). At the end of the experiment, participants completed the Toronto Alexithymia Scale^87^ (TAS-20) and the Interpersonal Reactivity Index^88^ (IRI) questionnaires. Scores on both questionnaires were in the normal range (TAS-20: *M* = 43.6, *SD* = 11.5 IRI: M = 98.75, SD = 8).

### Materials and apparatus

**Figure 4:**
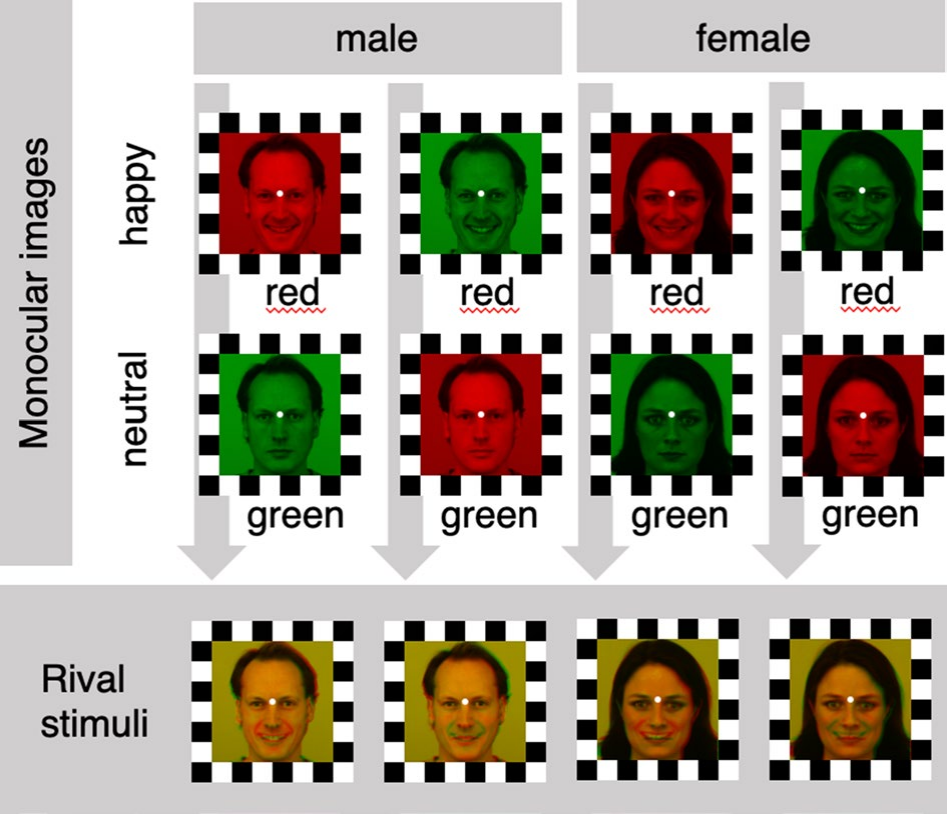
Monocular images and rival stimuli. Arrows indicate the resulting rival stimuli. Following the binocular rivalry experiment, the monocular stimuli in isolation were evaluated with regard to valence and arousal. A white 12-pixel fixation point and 40-pixel black and white squares frame were applied to the images to facilitate binocular fusion using GIMP (version 2.8.10, https://www.gimp.org). Monocular images contrast and luminance histograms were match by using Fiji (ImageJ 1.52c, https://fiji.sc). Original pictures (AM10NES, AM10HAS, AF01NES, AF01HAS) of facial expressions have been selected from the Karolinska Directed Emotional Faces set (KDEF; https://www.kdef.se/).

Visual stimuli were displayed using E-Prime 2.0 Software (version 2.0.10.242; Psychology Software Tools, Pittsburgh, PA) on a LG flatron F700B (Brightness: 85; Contrast: 90) 85 Hz monitor. Original stimuli were selected from the Karolinska Directed Emotional Faces^89^ and included one male (AM10) and one female (AF01) face each displaying a neutral and happy expression. Visual stimuli were presented covering 8 degrees of visual angle in height and width. Images were cropped with the software GIMP (version 2.8.10; see Figs. 1 and 4) 562 × 562 pixels centering the face in the middle of the square. Images of the same individual expressing the two emotions (happy and neutral) were superimposed and shifted by four pixels in order to facilitate the rivalry between two percepts. Happy and neutral faces of the same identity were analyzed and matched (in terms of contrast and luminance histograms) by using Fiji^90^ and MATLAB (version R2019; see Figs. 1 and 4).

### Procedure

Participants seated on a comfortable chair in a silent, temperature-controlled room. They were asked to place their head on a chin rest, with a distance of 70 cm from the screen. Before starting the experiment, the anaglyph filters (red/green) were set and participants were trained. The side of the color filters was counterbalanced across participants. During the experiment, participants were asked to focus on a fixation point located in the middle of the screen. The experiment consisted of one session of four blocks. Each participant performed two blocks where they could freely use their facial mimicry (“free mimicry condition”) and in other two blocks they were asked to hold a chopstick between their lips without using their teeth (“blocked mimicry condition”). The order of the two mimicry conditions was counterbalanced across participants. In each block, four rivalry stimuli were presented in a random order for a total of eight trials (twice per stimulus) in each block (see Fig. 4). Rivalry stimuli were presented for 15 s preceded by a 2-s fixation point and followed by a 3-s black screen. Participants were asked to code what they saw in real time by pressing one of three keys of the keyboard (“b”, “n”, “m”, these keys are adjacent to each other on the standard keyboard in this order from the left to the right). Participants were informed that on each trial they could see one of two faces, and that the appearance might change from one to the other during the trial. Coding instructions were presented before the beginning of the block; the order of the “b” and “m” keys, corresponding to the coding of the “happy” and “neutral” facial expression, was counterbalanced across blocks, while the “n” key always corresponded to the coding of a “mixed” percept. In the middle and at the end of each block a short break was recommended to the participant to reduce any fatigue. At the end of each mimicry conditions (i.e., two blocks) valence and arousal of each stimulus were measured respectively on a − 3/+ 3 and + 1/+ 7 scales.

### Data reduction

Firstly, for each trial we extracted the initial percept (IP), namely the first reported percept during the ongoing trial (neutral and happy facial expressions), in order to analyze whether initial percept frequencies changed as a function of the emotionality of the face and/or as a function of the mimicry manipulation. We further computed onset resolution time (ORT), namely the time to code the IP as a mean per emotion per subject. ORT log transformation was used for the analysis. We also computed cumulative times (CTs), as a measure of perceptual predominance, for mixed percept, neutral and happy facial expressions separately. That is, CTs were computed by summing the perceptual duration for each of mixed, neutral and happy percepts segments during a trial. Two participants did not code a total of three trials (Subject 9, one trial in the blocked mimicry condition; Subject 18, two trials in the free mimicry condition).

### Data analysis

Differences in stimuli rating for valence and arousal were assessed in separate analyses of variance (ANOVAs) and post hoc comparisons. Differences in mimicry (blocked and free) and IPs (happy, neutral) were assessed for ORT in analyses of variance (ANOVA) and post hoc comparisons. All post hoc comparisons are Bonferroni corrected for multiple comparisons. Differences in mimicry (blocked and free) and facial expression percept (happy, neutral and mixed) were assessed for CT in analyses of variance (ANOVA) and post hoc comparisons. All post hoc comparisons are Bonferroni corrected for multiple comparisons. In order to estimate the probability of the IP as a function of facial mimicry manipulation we applied a mixed-effects logistic regression model with IP (happy or neutral) explained by mimicry condition (blocked vs. free). Subjects were inserted as random effect with a varying intercept. The mimicry effect was evaluated as the odds ratio between free and blocked conditions.

## Data Availability

The dataset and analyses reported in this manuscript are available at Open Science Framework repository: https://osf.io/xk25b/.
